# Conceptualisation of community-based rehabilitation in Southern Africa: A systematic review

**DOI:** 10.4102/sajp.v72i1.301

**Published:** 2016-09-23

**Authors:** Vyvienne R. P. M’kumbuzi, Hellen Myezwa

**Affiliations:** 1 Physiotherapy Department, University of Malawi, Blantyre, Malawi; 2 Physiotherapy Department, University of the Witwatersrand, Johannesburg, South Africa

## Abstract

**Background:**

Community-Based Rehabilitation (CBR) has evolved over the last 30 years and now focuses on empowering persons with disabilities to access and benefit from a wide range of services. The evidence for CBR is frequently cited in the literature as being scanty and of poor quality.

**Purpose:**

We sought to determine how CBR is conceptualised and understood in the literature from Southern Africa. Our interest centred on to what extent the literature could inform policy makers and practitioners in the region.

**Methods:**

A systematic review of the literature from countries in Southern Africa guided by Population, Intervention/Phenomenon of Interest, Context and Outcome of Interest to the reviewer (PICO) was employed. This involved an extensive, internally valid and systematic search of electronic databases using specific keywords/subject heading combinations. Journal articles reporting on a description or objectives of CBR, published after 2006, and journal articles written in English of all types of studies were included. Data were charted according to the emergent themes. Two independent raters coded the emergent themes.

**Results:**

Nine from a possible 257 published articles were reviewed; four of these were programme evaluations. Themes describing CBR converged on community development and poverty reduction. Only one article referred to human rights. Training and supervision of CBR workers and education of the community about disability were frequently reported activities.

**Conclusion:**

In isolated cases, the literature is aligned to components of the CBR matrix. However, consistent with previous criticism of CBR, the literature is meagre, as is the evidence to inform policy makers and practitioners in southern Africa.

## Introduction

Community-based rehabilitation (CBR) was initiated in the mid–1980s by the World Health Organization (WHO) and over the years has evolved into a multi-sectoral strategy that empowers persons with disabilities to access and benefit from a wide range of services.

CBR is a strategy for enhancing the quality of life of people with disabilities (PWDs), improving service delivery by providing more equitable opportunities and social integration, and promoting and protecting their human rights (WHO 2011). Although the origins of CBR are rooted in facilitating primary rehabilitation in low-income countries (Helander 2007), it is now a response, in both developed and developing countries, to the need for adequate and appropriate rehabilitation services, to be available to a greater proportion of the disabled population. Its development and practice as a need-based approach has evolved from a bio-medical approach to a bio-psychosocial approach (Bury 2005; Finkenflugel, Wolfers & Huijsman 2005; Helander & Mendis 1991). In order to achieve its goals, CBR calls for full and coordinated involvement of all levels of society, community, provincial and national (Helander & Mendis 1991; Sharma 2007; ILO, UNESCO & WHO 2004).

The development of CBR was influenced by the definition of disability and the human rights movement. The joint position paper of the ILO, UNESCO and WHO (2004) outlines the evolution of the concepts of CBR such as disability and rehabilitation. There is recognition of the social model of disability; human rights; poverty reduction; inclusive communities; and the role of Disabled Peoples’ Organisations (DPOs) as educating all PWDs about their rights, advocating for action to ensure these rights, and collaborating with partners to exercise rights to access services and opportunities, often within CBR programmes. The UN Convention on the Rights of People with Disabilities (2006) sets out the legal obligations on states to promote and protect the rights of persons with disabilities. Participation of PWDs in social and economic development programmes is important to correctly identify specific needs, and to empower the individual. Full and effective participation and inclusion in society is recognised in the convention as: a general principle (article 3), a general obligation (article 4) and a right (articles 29 and 30).

The CBR guidelines (WHO 2010) capture the essence of participation of PWDs as a rights-based approach. These guidelines describe a CBR matrix (Table 1) that consists of five key development areas – health, education, livelihood, social and empowerment – and promote mainstreaming and empowerment of PWDs and their family members (WHO 2010).

**TABLE 1:** Community-based rehabilitation (CBR) matrix.

Health	Education	Livelihood	Social	Empowerment
Promotion	Early childhood	Skills development	Personal assistance	Advocacy & communication
Prevention	Primary	Self-employment	Relationships, marriage and family	Community mobilisation
Medical care	Secondary and higher	Wage employment	Culture and arts	Political participation
Rehabilitation	Non-formal	Financial services	Recreation, leisure and sport	Self-help groups
Assistive devices	Lifelong learning	Social protection	Justice	Disabled Peoples’ Organisations

*Source*: Community-based rehabilitation guidelines, WHO 2010

Each column includes five areas of activity, which are potentially part of CBR. The focus of the guidelines is mainstreaming and empowerment of PWDs and their family members, and sustainability of CBR (WHO 2010). The International Classification of Functioning, Disability and Health (ICF) underpin these guidelines in terms of defining disability (WHO 2001).

In many countries, CBR at the community level is part of an integrated community development programme that relies on the mobilisation of local resources (Sharma 2007; WHO 2010). The family of the disabled is the most important resource, supported by the community to provide the basic necessities of life and help the families to carry out rehabilitation. At the intermediate level, a network of professional support services should be provided by the government. Its personnel should be involved in the training and technical supervision of community personnel and should provide services and managerial support, and should liaise with referral services. Referral services are needed to receive those disabled people who need more specialised interventions than what the community can normally provide (Sharma 2007). At the national level, CBR seeks the involvement of the government in the leading managerial role (Helander 2007).This concerns planning, implementing, coordinating and evaluating the CBR system. This should be done in cooperation with communities, the intermediate level and the non-governmental sector, including organisations of disabled people.

An alternative term, ‘Disability Inclusive Development’, is now used to denote promotion of inclusion and making comprehensive healthcare, education and rehabilitation services available and accessible to people with disabilities (CBM 2015).

Against the backdrop of an evolving approach to CBR globally, a variety of government and non-government stakeholders involved in CBR, as well as implementation of CBR in variable political and social - cultural contexts, the collective understanding and scope of CBR among CBR practitioners in present day Southern Africa is unknown. The researchers undertook to develop an evaluation tool for CBR in Southern Africa. A preparatory phase to this work involved conceptualising CBR in the region in a three-part study, comprising: (1) a policy proof of concept, (2) a systematic review and (3) a description of on the ground experience from field visits. The purpose of this paper is therefore to determine the current conceptualisation of CBR in Southern Africa, by evaluating the literature available in the region.

## Methods

In this study, Southern Africa was defined as all countries belonging to the Southern African Development Community (SADC). These countries – Angola, Botswana, Democratic Republic of Congo, Lesotho, Madagascar, Malawi, Mauritius, Mozambique, Namibia, South Africa, Swaziland, Seychelles, Tanzania, Zambia and Zimbabwe, are located in the southernmost part of the African continent and share one or more borders with one, two or more of the other member states.

The SADC was established in 1980 in Lusaka, Zambia, as a loose alliance of nine majority-ruled states in Southern Africa, known as the Southern African Development Coordination Conference (SADCC).

The SADC strives for regional integration to promote economic growth, peace and security in the Southern African region. It aims to create common political values, systems and institutions among its 15 member states, to build social and cultural ties, and to help alleviate poverty and enhance the standard of living among a regional population of over 310 million.

### Procedure

A systematic review of the literature on the current conceptualisation of CBR in Southern Africa was undertaken. Southern Africa was selected because all countries in the region are implementing CBR in one form or another, and because the researchers are resident in this region and have a special interest in CBR in the region. Articles were identified by two independent research personnel, researcher 1 (VM) in Malawi, and the Wits University Librarian in South Africa. The review identified peer-reviewed journal articles from Angola, Botswana, the Democratic Republic of Congo, Lesotho, Madagascar, Malawi, Mauritius, Mozambique, Namibia, South Africa, Swaziland, Seychelles, Tanzania, Zambia and Zimbabwe published between 2006 and 2014. The review focused on peer-reviewed literature in order to select articles based on sound scientific methods as the basis for conceptualisation of CBR from the literature.

The following keywords were used: ‘community-based rehabilitation’, ‘disability’, ‘Southern Africa’ and ‘Southern Africa country’. The search strategy used various combinations of the search words ‘community-based rehabilitation of people with disabilities’, ‘programmes’, ‘programme descriptions’, ‘objectives’, ‘disability’, ‘Southern Africa’, ‘year’ (2006–2014). The step-by-step search strategy is shown in Table 2.

**TABLE 2:** Literature search strategy.

Initial search	Extensive search
Google Scholar and Pub Med to determine specificity and appropriateness of keywords	SCOPUS, HINARI, Pub Med (includes in-process citations), CINAHL, EMBASE, AJOL, DATAD
134 articles found	Disability and rehabilitation-specific electronic databases: http://www.asksource.info and CIRRIE: http://www.cirrie.buffalo.edu/
Direct searches and contact with respective organisations	Key databases – WHO Africa Region – Division of Non-communicable Diseases and WHO Disability and Rehabilitation (DAR) and literature from organisations active in CBR in Southern Africa, i.e., CBM, Handicap International and SIDA
Manual searches from different reference lists	Hand and reference searches

*Source*: Author’s own work

Articles meeting the following reliable criteria were selected for review:

All types of studies reporting on a description or objectives of a CBR programme(s) of people with disabilities in one or more of the countries in Southern Africa.

Literature published from January 2006 (publication of the United Nations Convention on the Rights of Persons with Disabilities [UNCRPD]) to the current date.

### Articles written in English

Letters to the editor, short editorials and publications without abstracts or summaries were excluded. Articles without abstracts were excluded because a review of the abstract was part of the procedure for selecting articles.

The above criteria were applied sequentially such that initial steps excluded publications that were: not reporting on CBR of PWDs, not about a country in Southern Africa and published prior to 2006, ultimately not meeting the inclusion criteria.

**FIGURE 1:**
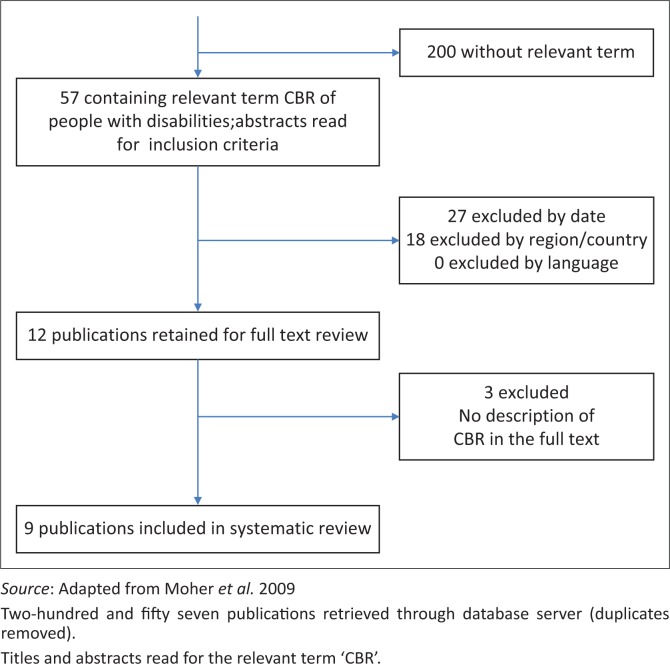
Number of articles selected for review.

Each subsequent step involved more detailed arbitration (Cleaver & Nixon 2014). The step-by-step article selection is shown in Figure 1.

The qualitative articles were assessed for methodological rigour using the Critical Appraisal Skills Programme (CASP) (Arksey & O’Malley 2005) by two independent reviewers (Table 3). The selection for the review was guided by PICO (Cooke, Smith & Booth 2012), as shown in Table 3. The background and introductory sections of the selected nine articles were read thoroughly. Data from the text which described CBR, its features and scope were extracted manually. Open coding and thematic content analysis was performed on this text. Emergent themes were tabulated to describe CBR in Southern Africa. This output was evaluated against the CBR matrix. A second researcher performed a check of the coding and evaluation of the themes against the CBR matrix.

**TABLE 3:** PICO for systematic review – conceptualisation of CBR in Southern Africa.

PICO elements	Description of PICO concept
Population – characteristics of the patient/population/condition or disease of interest	All CBR programmes in Southern Africa, reporting on people with disabilities (all types/multiple types). CBR was described broadly as all programmes where care of people with disabilities took place in the home/community by the family and or grassroots workers. For purposes of this review, ‘community-based rehabilitation’ was defined as any combination of a broad number of activities or interventions that can be included in the CBR matrix and are targeted at the rights, needs or inclusion of people with disabilities. Any report which self-identified itself as CBR was included in this review, except those that only described institution-based interventions. Likewise, if a project did not self-identify as CBR, it was not included (Bowers, Kuipers & Dorsett 2015).
Intervention – phenomenon of interest	Description and understanding of CBR. The words used to describe CBR.
Context	Geographical – Southern Africa. Both urban and rural, all types of foci – CBR orientation that is either medical or social; all types of disabilities targeted.
Outcome of interest to the reviewer	Published concepts. The words used to describe CBR, it’s nature, features and scope.

*Source*: Adapted from Cooke, Smith and Booth 2012

## Ethical considerations

Ethical approval was granted by the University of the Witwatersrand, Human Research Ethics Committee (Medical), South Africa (Clearance certificate number: M130953).

## Results

Table 4 charts the raw search output of the two independent searches by country and search engine, and the frequency of exclusions. An initial 479 articles were identified. Duplicate entries that arose from the two searches and the multiple engine searches were removed, leaving 257 articles for review; 12 met the inclusion criteria for a full text review. Figure 1 shows the refined output of the selection process.

**TABLE 4:** Search output.

Country/region	Search engine	Obtained	Excluded by				Retained
			Date	Relevance	Country	Language	
Southern Africa	AJOL	65	–	63	2	0	0
	Pub Med	14	4	4	3	0	1
	EBSCO	17	–	10	5	0	2
	WHO-DAR	10	5	–	2	0	0
	WHO Africa (NCDs)	73	2	39	6	0	0
Angola	AJOL	3	–	3	–	0	0
Botswana	Pub Med	1	1	–	–	0	0
	AJOL	7	–	7	–	0	0
	Google Scholar	6	–	6	–	0	0
DR Congo	Pub Med	0	–	–	–	0	0
	AJOL	15	–	15	–	0	0
Malawi	Pub Med	1	–	1	–	0	0
	AJOL	16	–	16	–	0	0
Madagascar	AJOL	3	–	3	–	0	0
Mauritius	AJOL	3	–	3	–	0	0
Mozambique	AJOL	8	–	8	–	0	0
Lesotho	AJOL	3	–	3	–	0	0
Namibia	AJOL	4	–	4	–	0	0
Seychelles	AJOL	2	–	2	–	0	0
South Africa	Pub Med	12	2	6	–	0	4
	AJOL	187	–	–	–	0	–
	Hand search	1	–	–	–	0	1
	Reference search	2	–	–	–	0	1
Swaziland	AJOL	3	–	3	–	0	0
Tanzania	All search engines	0	0	–	–	0	0
Zambia	Pub Med	1	1	–	–	0	0
Zimbabwe	Pub Med	5	5	–	–	0	0
	AJOL	18	–	11	–	0	2
**Total**		**479**	**27**	**200**	**18**	**0**	**12**

*Source*: Author’s own work

Only nine articles, published between 2008 and 2014, were reviewed from an initial search output of a possible 257. Four of these were programme evaluations: two were based on action research, two were original research papers and one was a reflective perspective of CBR. The majority (7) was papers on CBR in South Africa. The reviewed articles were published in various journals.

Table 5 shows the final selection of peer-reviewed articles that met the inclusion criteria and where the full text was reviewed.

**TABLE 5:** Full text reviewed articles.

Author (year)	Title	Journal	Country	Publication type	Final selection(yes/no)
Maart and Jelsma (2014)	Disability and access to health care – a community-based descriptive study.	*Disability and Rehabilitation*	South Africa	Descriptive, research	No description of CBR
Campbell (2011)	Power. Politics and rehabilitation in sub-Saharan Africa, from the personal to the political.	*Disability and Rehabilitation*	Sub-Saharan Africa	Reflective Perspective	Yes
Mji *et al*. (2009)	Realising the rights of disabled people in Africa: an introduction to the special issue.	*Disability and Rehabilitation*	Africa	Review	No description of CBR
Lorenzo and Joubert (2011)	Reciprocal capacity building for collaborative disability research between disabled people’s organisations, communities and higher education institutions.	*Scandinavian Journal of Occupational Therapy*	South Africa	Review	No description of CBR
Wasserman, de Villiers and Alan Bryer (2009)	Community-based care of stroke patients in a rural Africa setting.	*South African Medical Journal*	South Africa	Original research	Yes
Binken, Miller and Concha (2009)	The value of the service offered by the community rehabilitation worker.	*South African Journal of Occupational Therapy*	South Africa	Programme evaluation	Yes
Kudzai and Ganga (2010)	An evaluation of Community Based Rehabilitation of persons with special needs in Zimbabwe.	*African Journal for the Psychological Study of Social Issues*	Zimbabwe	Programme evalua-tion	Yes
Dawad and Jobson (2011)	Community-based rehabilitation programme as a model for task shifting.	*Disability and Rehabilitation*	South Africa	Original qualitative research	Yes
Chappell and Johnannsmeier (2009)	The impact of community based rehabilitation as implemented by community rehabilitation facilitators on people with disabilities, their families and communities within South Africa.	*Disability and Rehabilitation*	South Africa	Impact evaluation	Yes
Rule (2008)	CBR students’ understanding of the oppression of people with disabilities.	*South African Journal of Occupational therapy*	South Africa	Action research	Yes
Grandisson *et al*. (2014)	Community-based rehabilitation programme evaluations: lessons learned in the field.	*Disability, CBR and Inclusive Development Journal*	South Africa	Programme evaluation	Yes
Rule (2013)	Training CBR Personnel in South Africa to contribute to the Empowerment of Persons with disabilities.	*Disability, CBR and Inclusive Development Journal*	South Africa	Action research	Yes

*Source*: Author’s own work

Table 6 shows the performance of the different articles on the 10 CASP questions. Two articles that were not assessed were ineligible for assessment using the CASP. Most articles did not consider the relationship between the researcher and the participants (Question 6). All articles were however judged to be valuable (Question 10).

**TABLE 6:** Table showing performance of the different articles on the 10 Critical Appraisal Skills Programme (CASP) questions.

Article	Response	1	2	3	4	5	6	7	8	9	10	Total
Campbell(2011)	Yes	-	-	-	-	-	-	-	-	-	-	Not assessed
	Can’t tell	-	-	-	-	-	-	-	-	-	-	
	No	-	-	-	-	-	-	-	-	-	-	
Wasserman *et al*. (2009)	Yes	-	-	-	-	-	-	-	-	-	-	Not assessed
	Can’t tell	-	-	-	-	-	-	-	-	-	-	
	No	-	-	-	-	-	-	-	-	-	-	
Binken *et al*. (2009)	Yes	✓	✓	✓	✓	✓	-	-	✓	✓	✓	8
	Can’t tell	-	-	-	-	-	✓	-	-	-	-	
	No	-	-	-	-	-	-	✓	-	-	-	
Kudzai and Ganga (2010)	Yes	-	✓	-	-	✓	-	-	-	✓	✓	4
	Can’t tell	-	-	✓	✓	-	-	-	-	-	-	
	No	✓	-	-	-	-	✓	✓	✓	-	-	
Dawad and Jobson (2011)	Yes	-	✓	✓	✓	✓	-	-	✓	✓	✓	8
	Can’t tell	-	-	-	-	-	-	✓	-	-	-	
	No	✓	-	-	-	-	✓	-	-	-	-	
Chappell and Johnannsmeier (2009)	Yes	✓	✓	✓	✓	✓	-	✓	✓	✓	✓	9
	Can’t tell	-	-	-	-	-	✓	-	-	-	-	
	No	-	-	-	-	-	-	-	-	-	-	
Rule (2008)	Yes	✓	✓	✓	✓	✓	✓	✓	✓	✓	✓	10
	Can’t tell	-	-	-	-	-	-	-	-	-	-	
	No	-	-	-	-	-	-	-	-	-	-	
Grandisson *et al*. (2014)	Yes	✓	✓	✓	✓	✓	✓	-	✓	✓	✓	9
	Can’t tell	-	-	-	-	-	-	✓	-	-	-	
	No	-	-	-	-	-	-	-	-	-	-	
Rule (2013)	Yes	✓	✓	-	✓	✓	-	-	✓	✓	-	6
	Can’t tell	-	-	✓	-	-	-	-	-	-	-	
	No	-	-	-	-	-	✓	✓	-	-	✓	

*Source*: Author’s own work

### Concepts revealed in description of CBR

The emerging CBR concepts of the reviewed articles centred on power and politics in CBR programmes, access to rehabilitation and task shifting as well as the impact of CBR. An underlying but minor theme was the social construct of disability and social and cultural patterns of behaviour of communities towards PWDs. One article highlighted training of personnel for CBR.

All the articles referred to disability in general, and no special disability categories, gender or age groups were defined. The dominant descriptions of CBR are presented in Table 7.

**TABLE 7:** Descriptions of CBR.

CBR concept – What is CBR? What is involved? What does it aim to achieve?	Concept processes Who is involved? How is CBR done?	Source
Partnership between those with more skills and those with local skills and knowledge; outreach; large-scale transfer of knowledge and skills, used in community resources.	PWDs and family – pi-votal role and combined efforts. DPOs; Community involvement, coordinated involvement of all levels of society.	Campbell (2011); Kudzai and Ganga (2010)
Disability is a social construct, CBR a social model; poverty reduction.	Professional = facilitator/consultant/director.	Dawad *et al*. (2011); Binken *et al*. (2009)
Equalisation of opportunity; social inclusion.	3-tiers – coordinator, local supervisor, families and communities.	Rule (2008); Chappell *et al*. (2009)
Structural – remove social, economic and institutional barriers; include human agency – individuals and independently develop self-esteem and self-confidence; empowerment.	Volunteers have key input. Three levels of personnel – grassroots, mid-level and professionals.	Rule (2013); Dawad *et al*. (2011); Chappell (2009)
Raise disability awareness; advocate and lobby for disability rights.	Mid-level work prominent; CRW/therapy assistant is responsible for rehab at community and household levels.	Rule (2013)
Community development; poverty reduction, income-generating projects, support and training of community workers.	Support from national level poli-cy, coordination and resource. Strengthening of referral systems.	Chappell *et al*. (2009)
Physical rehabilitation – secondary prevention, early intervention, support needs, medical suppliers, home programme training of PWDs, parents of CWDs and families.	Coordinated multi-sectoral ap-proach. Many different organisations/civil society and the church.	Rule (2013); Grandisson *et al*. (2014); Dawad *et al*. (2011); Binken *et al*. (2009); Chappell (2009)
Enhance independence and quality of life PWDs.	State and civil society must dismantle the barriers.	Rule (2013); Grandisson *et al*. (2014); Dawad *et al*. (2011); Binken *et al*. (2009); Chappell (2009)
Schooling, self-help groups, pre-academic skills, occupational skills, behaviour/attitude modification, psychosocial skills and counselling.	Closely related to PHC. Use technology close to local experience.	Dawad *et al*. (2011); Wasserman *et al*. (2009); Kudzai and Ganga (2010)

*Source*: Author’s own work

The descriptions of CBR were further analysed by aligning each description to the components of the CBR matrix. The results are shown in Table 8.

**TABLE 8:** Alignment of CBR descriptions to the CBR matrix.

CBR concept – What is CBR? What is involved? What does it aim to achieve?	CBR matrix column	CBR matrix sub-category
Partnership between those with more skills and those with local skills and knowledge; outreach; large-scale transfer of knowledge and skills; use of community resources.	Livelihood	Skills development
Disability is a social construct CBR a social model; Poverty reduction.	Livelihood	
Equalisation of opportunity; social inclusion.	Empowerment	Advocacy & communication
Structural – remove social, economic and institutional barriers; includes human agency – individuals & independently develop self-esteem and self-confidence; empowerment.	Empowerment	Advocacy & commu-nication Political participation
Raise disability awareness; advocate and lobby for disability rights.	Empowerment	Advocacy & commu-nication Community mobilisation
Community development; poverty reduction, income-generating projects; support and training of community workers.	Livelihood	
Physical rehabilitation – secondary prevention, early intervention, support needs, medical suppliers, home programme training of PWDs, parents of CWDs and families.	Health	Prevention Medical care Rehabilitation
Enhance independence and quality of life PWDs. Schooling, self-help groups, pre-academic skills, occupational skills, behaviour/attitude modification, psychosocial skills and counselling.	Education Empowerment	Early child-hood Non-formal Self–help groups

*Source*: Author’s own work

There was convergence of what CBR is around empowerment, livelihood, health and education. Notably only one article from 2013 mentions human rights.

In terms of who is involved in CBR, the literature placed the person with a disability and their family at the centre of CBR. There was also agreement on community involvement, with specification of certain sectors of the community, for example, civil society, the church and DPOs. A three-tier system of grass roots workers, mid-level rehabilitation workers and professionals were responsible for the delivery of CBR, whilst the government was responsible for policy formulation, coordination and provision of resources. There was remarkable consensus around who is involved in CBR, although only the roles of the government, civil society and middle-level community rehabilitation workers were elaborated.

Common CBR activities included training and supervision of different levels of CBR workers – family, CRW, parents of children with disabilities (CWDs) (most); and educating the community about the needs of PWDs and the prevention of disabilities.

Information relating to how CBR could be delivered identified the following:

- removal of barriers – structural/functional in environment (physical buildings, legal)
- raising disability awareness; advocacy and lobbying for disability rights
- use of local resources
- building skills/capacity.

## Discussion

To our knowledge, this is the first review that focuses on a proof of concept for CBR in Southern Africa. Information that provides evidence of our understanding of CBR in Southern Africa remains scanty and outdated. The search for literature on CBR in the region yielded remarkably little information that could be representative of the region. Cleaver and Nixon (2014) reported similar results in a scoping review focusing on the characteristics of peer-reviewed literature on CBR in low- and middle-income countries published in English from 2003 to 2012. Most of the reviewed articles in this review were from South Africa. We attribute this finding to the existence of many universities in South Africa, and only South Africa (University of Cape Town, Stellenbosch University, University of KwaZulu-Natal, University of Pretoria, University of Limpopo, and University of the Witwatersrand) in the region offers graduate and research programmes in the rehabilitation professions (physiotherapy, occupational therapy and speech and language therapy), and are therefore likely to attract research funding under these established programmes.

Only three reviews of CBR have previously been done – the first by Finkenflugel *et al*. (2005) sought the evidence base for CBR, and from 128 articles included in the final review from the developing world published between 1978 and 2002 concluded that ‘key aspects that are at the heart of the definition of CBR (i.e. the involvement of PWDs, community members and rehabilitation workers, and the use of local resources) are among the less-researched aspects.’ In our review, these descriptions of CBR feature, in that the involvement of PWDs and their families as well as the community- and mid-level rehabilitation workers and professionals are listed as those through whom CBR is delivered. The second review was a scoping review of 10 years (2003–2012) of published literature on CBR by Cleaver and Nixon (2014). The purpose of this scoping review was to identify the characteristics of peer-reviewed literature on CBR in low- and middle-income countries published in English from 2003 to 2012. The third review was by Iemmi *et al*. (2015). This recently published review assessed the impact of CBR on the lives of PWDs and their carers in low- and middle-income countries, and included 15 studies from 1976 to 2012. All these previous reviews had a different focus to the current review. All three reviews state concerns over the methodological quality of the studies reviewed. Our review supports these concerns. The strength of the evidence base in Southern Africa is not impressive, for example, even though four of the nine articles reviewed were programme evaluations, only one described the programme objectives. This makes it difficult to know the basis for the evaluation.

The description of CBR in the literature from Southern Africa contains aspects of the key components of the CBR matrix – health, education, livelihoods and empowerment. It recognises that CBR is a social model but does not contain any of the sub-categories of the social component. This review sought articles published since 2006, that is, prior to the publication of the CBR guidelines and the CBR matrix. The descriptions of CBR also appear relatively uninfluenced by the publication of the UN Convention on the Rights of People with Disabilities (2006), as evidenced by only one article describing CBR from a human rights perspective (Campbell 2011). The descriptions of CBR in the literature pick up a lot more on the contents of the Joint Position paper of the ILO, UNESCO and WHO (2004) which outlines the evolving concepts in CBR, in particular, the social model of disability, the poverty reduction and the role of DPOs. It seems therefore that the descriptions of CBR in the literature from Southern Africa have moved little from the 2004 era. We had expected the descriptions of CBR to lean more towards components of the CBR matrix contained in the CBR guidelines (2010) as well as towards a more rights-based approach contained in the UN Convention on the Rights of People with Disabilities (2006). The focus on the social model and poverty reduction may be attributed to the reciprocal relationship between disability and poverty. Campbell (2011) writes that sub-Saharan Africa is beset with ‘many challenges, including endemic poverty’. Most of the articles cited in this review are about the rural parts of Africa where these widespread social and economic challenges prevail to a greater degree.

The researchers premised the choice of methodology on the fact that systematic reviews have become a cornerstone of the evidence-based practice and policy movement. The synthesis and use of qualitative evidence for informing policy making and professional practice has been developed as a specific methodology for searching for, and appraising and synthesising, findings of primary studies. Policy makers and practitioners reading the CBR literature from the region could derive a picture of what CBR entails but would not be able to determine the specific services and opportunities that PWDs should be able to access as this is relatively silent. Further, the full description of CBR is not contained in any one article; they would have to read a spread of the literature to obtain the picture of CBR.

In spite of the few published studies contained in this review, the authors are aware that there are numerous CBR programmes going on in Southern Africa. In fact, each of the 15 countries is implementing CBR in one form or another, but this is not documented in the peer-reviewed literature.

### Implications for practice

The evidence in terms of descriptions of CBR in the literature from Southern Africa is scanty, scattered and outdated. Substantially more research is required that focuses on practices that have leanings towards the more recent global instruments that describe CBR, such as the UNCRPD and CBR guidelines. Most of the studies reviewed were qualitative studies, whose methodological rigour varied widely, ranging from a score of 4 to 10 on the CASP. No article had a randomised control study design to show the efficacy of CBR interventions, thereby placing the evidence on the weaker side. Practitioners in the region also need to cultivate a culture of documenting their practices in the peer-reviewed literature. In its current state, the literature does not provide sufficient evidence to guide practice or policy.

### Strengths and limitations

The researchers strengthened the search process by searching across multiple electronic databases, and also including hand and reference searches. An increased number of suitable articles could have been obtained had social science electronic databases been included. The initial volume of literature was expensive and therefore at the time of the literature search, social science databases were not included. On completing the analysis, only nine articles were suitable. Future research should include a search of social science databases as this would give a comprehensive picture.

## Conclusion

CBR is described in terms of being a social model that aims to reduce poverty among PWDs. It is also described in terms of physical rehabilitation and the enhancement of the quality of life of PWDs through schooling and occupational skills. The PWD and the family are at the centre of CBR and are supported by grassroots workers, middle-level rehabilitation workers and professionals. Future research needs to address the rights of PWDs and the social components of the CBR matrix.
